# Leveraging AI to Drive Timely Improvements in Patient Experience Feedback: Algorithm Validation

**DOI:** 10.2196/60900

**Published:** 2025-07-10

**Authors:** Mustafa Khanbhai, Catalina Carenzo, Sarindi Aryasinghe, David Manton, Erik Mayer

**Affiliations:** 1 iCARE Digital Collaboration Space & Secure Data Environment, NIHR Imperial Biomedical Research Centre, 1a Sheldon Square, London, W2 6PY, United Kingdom, 44 20 7589 5111; 2 Department of Surgery & Cancer, Faculty of Medicine, Imperial College London, London, United Kingdom; 3 Imperial College Healthcare NHS Trust, London, United Kingdom

**Keywords:** patient experience, quality improvement, free-text analysis, machine learning, artificial intelligence, patient feedback, friends and family test, natural language processing, algorithm, health informatics

## Abstract

**Background:**

Understanding and improving patient care is pivotal for health care providers. With increasing volumes of the Friends and Family Test (FFT) data in England, manual analysis of this patient feedback poses challenges for many health care organizations. This underscores the importance of automated text analysis, particularly in predicting sentiments and themes in real time.

**Objective:**

Leveraging machine learning and natural language processing, this study explores the utility of a supervised algorithm to systematically test and refine the algorithm’s cross-contextual performance in diverse health care settings, addressing variations in population characteristics, geographical locations, and care settings, ultimately driving improvements based on patient feedback.

**Methods:**

The text analytics algorithm initially developed in a large acute trust in London was further tested in 9 health care organizations with diverse care settings across England. These trusts varied in technical capacity and resource, population demographics, and FFT free text datasets. Testing and validation of the algorithm were performed, including manual coding of a subset of retrospective comments. Technical infrastructure, including coding environments and packages for algorithm testing and deployment, was optimized. The algorithm was iteratively trained using bag of words from anonymized data, tailored to accommodate contextual variations, and tested for change in algorithm performance while simultaneously rectifying issues identified.

**Results:**

The algorithm demonstrated satisfactory overall accuracy (>75%) in predicting themes and sentiments embedded within free-text responses across a variety of care settings and population demographics. While the algorithm yielded strong and reusable models in relatively stable environments, such as adult inpatient care settings, the initial accuracy was notably lower in organizations providing services such as pediatrics and mental health. However, the accuracy of our algorithm significantly improved when individual trust coding templates were applied. Thematic saturation was reached after the fifth organization was recruited, and no further coding was required for the last 4 organizations. Subsequently, a framework and pipeline for deployment of the algorithm were developed to provide a standardized approach for implementation and analysis of FFT free text, ensuring ease of use.

**Conclusions:**

This study represents a significant step forward in leveraging free-text FFT data for valuable insights in diverse health care settings through the testing and development of a robust supervised learning text analytics algorithm. The disparity in some care settings was anticipated, given that the lexicon and phraseology used was inherently different from those prevalent in adult inpatient care (where the algorithm was developed). However, these challenges were addressed with further coding and testing. This approach enhanced the accuracy and reliability of the algorithm, encouraged inter- and intraorganizational collaboration, and shared learning.

## Introduction

Insights into patients’ experience of their care provision are critical for health care providers delivering patient-centered care and recognized as integral to ensuring safe and high-quality care [1]. By the end of 2019, the national survey for patient experiences in the United Kingdom, known as the Friends and Family Test (FFT), had accumulated 75 million pieces of feedback [2]. This number continues to grow by about 1.3 million every month, making it the largest source of patient opinions worldwide. However, manually analyzing this vast amount of data requires a considerable resource, something that many health care organizations struggle to provide [3,4]. In recent years, there has been an ever-clearer desire for improvement to arise from patient feedback rather than just focusing on response rates, but most health care organizations are constrained from doing so due to a lack of analytical expertise [5].

Automated text analysis by artificial intelligence (AI) and its component natural language processing (NLP) predicts sentiments and themes within free-text responses in real time and provides a useful opportunity for better integrating patient experience feedback into everyday health care delivery [4]. NLP algorithms offer a practical solution to decode the “why” behind patient narratives, and through their ability to process large volumes of data accurately, these patient-derived insights can inform “business as usual” within health care organizations [6,7]. Recent advances in NLP, particularly the development of large language models and transformer-based architectures such as BERT [8] and GPT [9], have significantly improved the analysis of patient feedback in health care. These models replace traditional hand-coded, framework-based categorization [10] methods by automating the classification of concerns and providing decision-makers with real-time, comprehensive summaries of emerging themes. Large language models, through multi-label text classification [11], enable the extraction of deeper insights from patient experience comments, enhancing the understanding of the patient journey and informing actions that can improve care. Transformer-based models such as BERT [8,12] and GPT excel at capturing complex linguistic patterns, context, ambiguity, and sentiment, making them particularly well-suited for the nuanced task of sentiment analysis in health care. These advancements allow for more accurate, context-aware analysis of patient feedback, driving more actionable insights and supporting continuous improvements in patient care.

BERT and GPT models are large and resource-intensive [13]. They require significant computational power and memory, which may not be feasible in all health care settings, especially those with limited information technology (IT) infrastructure. This can make them less practical for real-time applications or for use in smaller organizations with fewer resources. Due to their size and complexity, transformer models can be slower to process compared to more lightweight models such as support vector machines (SVMs). In real-time health care applications where speed is crucial for timely interventions, this delay may be a significant drawback.

We have previously demonstrated the benefit of applying NLP using SVM to free-text FFT data to deliver patient-driven quality improvement in a single institution [14]. This study demonstrates the broader implementation and iteration of this algorithm in different hospital settings in England that use FFT to capture patient feedback.

## Methods

### Study Design

The implementation of the FFT free-text algorithm demands thorough testing to ensure robustness, accuracy, and applicability. To iterate the algorithm for deployment in other health care services, we first selected health care organizations that would incorporate geographical variables, patient demographic variables, and different care settings, that is, adult inpatient, primary care, community, pediatrics, and mental health.

The objectives of this study were to identify technical capabilities for algorithm deployment in the included health care settings, identify variation in user needs across different service settings and geographies, and iterate the algorithmic process and deploy a reproducible algorithm that can be implemented in different services.

### Trust Recruitment

**Figure 1.**
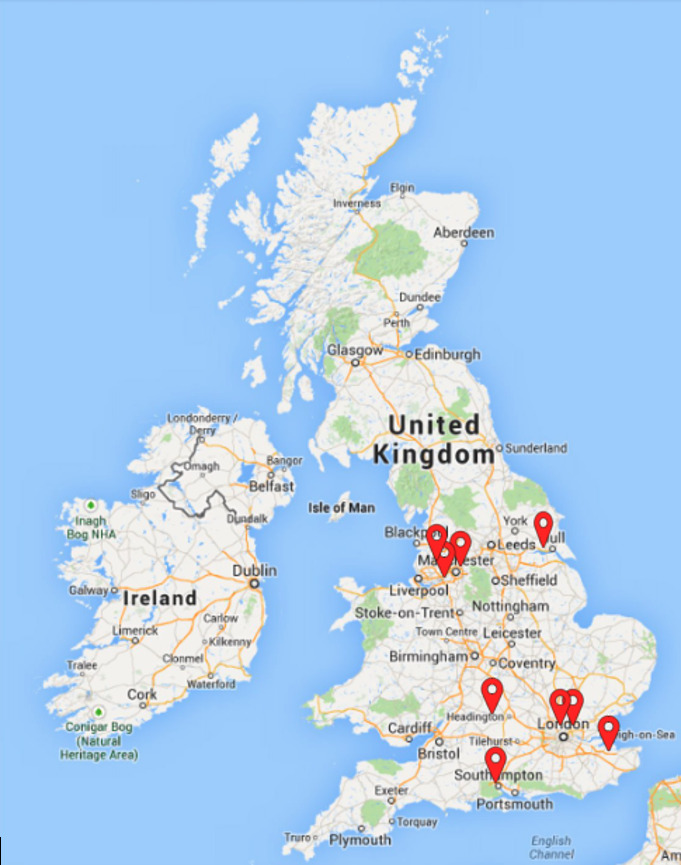
An overview of the geographical distribution of the 9 recruited trusts. Copyright owned by Imperial College Healthcare NHS Trust, 2025. All rights reserved.

A steering group consisting of lay representatives and patient experience experts was formed to guide the strategic direction of this study and help in recruitment through various patient experience networks including the National Insight Network set up by National Health Service (NHS) England, the Q Community established by the Health Foundation, Advancing Quality Alliance, and the Heads of Patient Experience Network. An FFT review capability questionnaire (Section 1 in Multimedia Appendix 1) was sent to interested organizations. This allowed this study’s team to identify organizations based on 3 key metrics: patient experience engagement, digital readiness or IT infrastructure, and quality improvement involvement. To ensure that the selected trusts possess the necessary infrastructural and organizational qualities to contribute effectively to the research objectives while adhering to data regulatory standards, a capacity and capability assessment was required. Only those trusts that were able to confirm capacity and capability through their Research and Development department were finally recruited, as shown in Figure 1.

### Ethical Considerations

This study received Health Research Authority and Health and Care Research Wales approval (20/HRA/5924). No patient data were collected, and trusts were anonymized in the analysis. Written consent was sought from members of the steering group and the individual hospital trust project team, and with further written consent, their contribution has been acknowledged in the Acknowledgments section.

### Preimplementation

#### IT Scoping Tool

To work collaboratively, we identified the needs of all participating organizations from a patient experience digital readiness standpoint. This was facilitated through previous work by this study’s team funded by NHS England Insight and Feedback Team [15], where a scoping tool was developed to identify key contacts, FFT data collection, SQL Server database, handling and management, infrastructure capacity, that is, on-premise data center or cloud computing, integrated development environment and coding language expertise, and data visualization software (Section 2 in Multimedia Appendix 1).

#### Data Access

To ensure that participating organizations were fully supported by the project team, a Data Protection Impact Assessment (DPIA) was completed with each organization. DPIA is a systematic process designed to identify and minimize the associated data protection risks. The purpose of a DPIA in our study was to assess the impact of processing data and to ensure that the principles of data protection, such as those outlined in regulations such as the General Data Protection Regulation were adhered to.

#### Adaptation of Coding Script

To achieve transferability of the SVM supervised machine learning (ML) algorithm and test against different service settings and NHS provider organizations, the functionalities previously developed [7] were recoded using Python (Python Software Foundation), given the popularity of using this coding platform in most NHS trusts. Python is an interpreted high-level general-purpose programming language. The model was developed with Python (version 3.8.10) with the following free Python libraries; scikit-learn≥0.22.1 [16] (an ML library that provides relevant ML packages required for classification), Natural Language Toolkit (NLTK)≥3.4.4 (a suite that contains libraries and programs to make machines understand human language), and Pandas≥0.25.3 (a library for data manipulation and analysis). These requirements were then translated into a requirements.txt file for organizations to deploy the algorithm. SVM was used as it is the most common classifier widely used for document classification, which consistently yields good classification performance [10].

#### Updating Training Coding Template

All FFT free-text comments were coded based on the NHS Patient Experience Framework (Section 3 in Multimedia Appendix 1). This framework incorporates 8 key themes that outline those elements that are critical to the patients’ experience of NHS services [17]. In the previous work [7], three additional themes were added: “unclassified” (too many typographical errors to discern meaning), “general” (eg, NHS is great, everything), and “staff” (comments relating to staff). From the NHS England funded work [15], it was observed that there was an overlap of comments between the theme “staff” and other themes such as “respect for patient-centered values, preferences, and expressed needs,” “emotional support,” and “physical comfort.” This is because often a staff member was involved in the delivery of the care as it related to the other themes. The theme “staff” was removed, and all comments coded as “staff” were recoded to develop a concise coding template. Therefore, 10 themes were selected for final analysis, and a coding pack (Section 4 in Multimedia Appendix 1) was developed with detailed guidance. Furthermore, we iterated the initial algorithm from our previous work which was developed using 2 follow-up free-text questions, that is, “What did we do well?” and “What could we do better?” by combining and shuffling the training data from both questions to develop a streamlined SVM-supervised ML algorithm for theme and sentiment prediction.

### Implementation

#### Overview

A pipeline was constructed for the SVM model in Python for text classification and sentiment analysis, consisting of several steps, including text preprocessing, feature engineering, TFIDF (term frequency-inverse document frequency) transformation, and model training and testing.

#### FFT Data Retrieval

SQL server was used to both extract the FFT data and supplementary free text for algorithm imputation, and to store the output of the algorithm. Pyodbc≥5.0.1 Python library was required to connect to the individual organizations’ SQL server. SQL’s combination of ease of use, flexibility, and capabilities make it useful for storing, managing, querying, and analyzing datasets and is widely used in health care organizations.

#### Establishing Ground Truth

In supervised learning algorithms, ground truth data is critical to training and updating an algorithm [10]. Therefore, more annotated data was required to improve the performance of the algorithm. The coding pack (Section 4 in Multimedia Appendix 1) developed was provided to the participating organizations’ patient experience teams to make coding more feasible. The coders were a combination of the patient experience team, clinical staff, and trust lay representatives. Two coders from the team completed manual coding individually using a 500-stratified sample to calculate interannotator reliability and understand the interpretation of themes and sentiment. Disagreements in coding were reviewed, and theme and sentiment were finalized to create a master coding template in a .csv file. This template was populated with data from all 9 organizations.

#### Preprocessing

Preprocessing of textual data is the first essential step in the processing of text and has been proven to improve text classification models’ performance by standardizing the text before it is presented to the classification algorithm [10]. NLTK for Python was used for preprocessing and data analysis, which includes tokenization of the comments and removal of stop words (eg, the, was, is, etc), punctuation, numbers, and special characters from the comments. The resulting words were represented as a bag of words (BoW) or corpus. Given that the average length of FFT free-text comments tends to be short, and the context is domain-specific, that is, patient experience, this BoW approach was used rather than word embedding.

#### Feature Engineering

Feature engineering is the process of transforming data into features or attributes that better represent the underlying structure of the data [10]. To identify the key features of the data, we used a statistical measure, TFIDF, used in the fields of information retrieval [18] and ML, that can quantify the importance or relevance of words in a comment among a collection of comments [19].

#### Determining Accuracy

To understand if further coding was required, the accuracy for theme and sentiment was calculated using a 500-stratified sample of manually coded comments from each of the individual organizations. The accuracy is defined as a metric used in classification to measure the percentage of accurate predictions [10]. It is also defined as the ratio of several correct predictions by the algorithm to the total number of predictions. In other words, the ratio of true positives (TPs) and true negatives (TNs) to all positive and negative observations (where FPs and FNs are false positives and false negatives, respectively): accuracy=(TP+TN)/(TP+TN+FP+FN). The acceptable threshold for overall accuracy for theme and sentiment for a supervised algorithm using patient feedback was determined at 75%. The 75% accuracy threshold for theme and sentiment classification was established based on the work of this study’s group [10] and previous studies in the literature [20,21]. Specifically, the threshold reflects the balance between achieving an acceptable level of accuracy and the complexity of interpreting nuanced patient feedback using NLP and ML techniques. Therefore, the 75% accuracy benchmark was identified as a reasonable trade-off between high-quality model performance and the inherent challenges in processing and classifying subjective feedback from patients.

The accuracies yielded by the models on different ranges of hyperparameters were compared. For SVM, both L1 and L2 regularization, variance tolerance values for the stopping criteria, and different values for the inverse of regularization strength, C, were compared. As regularization has a known role in reducing overfitting, the hyperparameters were chosen for tuning to identify the amount and type of regularization and early stopping criteria that are suitable for the classification tasks. After this, the optimal hyperparameters were found by GridSearchCV from the scikit-learn library. A 10-fold cross-validation was used to search to select the best hyperparameter combinations from specified ranges of hyperparameters, which were used to retrain the model on the training set and to predict on the test set [10]. This allowed us to establish the accuracy but also use the coded comments as a template to improve the accuracy by a further round of recoding if needed, until the accuracy was at or above 75%, that is, reached thematic saturation.

#### Algorithm Deployment

The master coded template data was then serialized using the Python pickle library. This module is used for serializing and deserializing objects into Python format text strings, which are called pickles and can be created by Python code or sent from other Python programs. This Python pickle is easily carried over the network and can be validated without knowing the details of the received object. This enables the preservation and sharing of the ML models, allowing users to reload pretrained models, significantly reducing the need for lengthy retraining. The files that were stored in the environment were as follows: Feature_sentiment.pkl, Feature_theme.pkl, Sentiment_classifier.pkl, Theme_classifier.pkl, Tfidftransformer_sentiment.pkl, and Tfidftransformer_theme.pkl.

#### Establishment of a Community of Practice

In the pursuit of ensuring the correct sustainability of the algorithm, a strategic initiative was undertaken through the establishment of a technical community of practice (CoP). This community served as a conduit for regular interaction and collaboration among the leaders responsible for overseeing the algorithmic maintenance across different trusts. Recognizing the complexity and potential challenges associated with deploying advanced algorithms, quarterly meetings were instituted as a core component of the CoP framework. These meetings provided a structured way for the leads of each site to convene, fostering an environment conducive to the exchange of critical insights, best practices, and lessons learned.

## Results

### Overview

Nine trusts were recruited from a range of care settings, sociodemographic and geographical areas. Trusts were added to this study sequentially, one by one, as part of the recruitment process. None of these organizations had a pre-existing automated process for analyzing their FFT free-text responses.

### Identifying Free-Text Comments With FFT Survey for Analysis

In the preimplementation scoping exercise, it was observed that among the participating organizations, trusts A and D incorporated 3 follow-up free-text questions in their FFT survey. In contrast, all other trusts included only 1 follow-up question (Table 1). The question with the highest number of comments in trust A was analyzed. The organizations provided a variety of services, including pediatrics, inpatient and outpatient, and community and mental health, thereby facilitating testing and iteration of the algorithm for use in all care settings. Furthermore, most of the organizations 88.9% (8/9), opted to house and deploy the algorithm on premises, and the rest on cloud 11.1% (1/9).

**Table 1.** An overview of the variation in FFT^a^ free-text data, service setting, and environment favored to deploy the machine learning algorithm in 9 NHS^b^ organizations in England. Version deployed refers to the version of the iterative addition of words to the BoW^c^ model, used to improve model performance.

Trust	Service coverage	Model deployment environment	Number of free-text columns	Free-text questions	Version deployed
A	Community and mental health	On premises	3	Please, can you tell us why you gave your answer?^d^ Please tell us at least one thing that went well. Please tell us at least one thing we could do better.	Version 0
B	Acute and inpatient	Cloud	1	Please, can you tell us the main reason for the score that you have given?	Version 1
C	Acute and inpatient	On premises	1	Please, can you tell us why you gave your answer and what we could have done better?	Version 2
D	Pediatrics	On premises	3	FFT rating description^d^ Write what you think was good. Write what you think was bad.	Version 3
E	GP^e^ or community	On premises	1	Please, can you tell us why you gave your answer?	Version 3
F	Acute and inpatient	On premises	1	Can you tell us why you gave that response?	Version 3
G	Acute and inpatient	On premises	1	Please, can you tell us why you gave your answer and what we could have done better?	Version 3
H	Acute and inpatient	On premises	1	What was good about your care, and what could be improved?	Version 3
I	Acute and inpatient	On premises	1	Please, can you tell us why you gave your answer?	Version 3

a FFT: Friends and Family Test.

b NHS: National Health Service.

c BoW: bag of words.

d The free text from this question was used in the algorithm testing, given the highest responses compared to the other 2 questions.

e GP: general practitioner.

### Variation in Patient Experience Themes

To understand the variation in themes, if any, of FFT free-text responses in the 9 organizations, we looked at the count of comments from the FFT free-text responses based on the NHS patient experience framework. This was extracted from the output after initial model deployment for the period of January 2022 to January 2023, as demonstrated in Table 2. The highest count of themes was “respect for patient-centered values” followed by “general.” The themes with the lowest count were “welcoming the involvement of friends and family” followed by “transition and continuity.” There were no demonstrable trends in the other patient experience framework themes.

**Table 2.** Distribution of count of themes based on the NHS^a^ patient experience framework from 9 NHS organizations in England, extracted from the output of the algorithm.

Trust	FFT^b^ comments provided per NHS Patient Experience Framework themes (%)									
	0^c^	1^d^	2^e^	3^f^	4^g^	5^h^	6^i^	7^j^	8^k^	9^l^
A	9	26	4	10	6	6	0	2	24	12
B	8	35	2	14	6	5	2	2	11	16
C	6	23	3	13	8	10	1	2	8	26
D	15	17	9	10	6	5	0	2	16	20
E	22	25	2	11	5	5	0	1	18	12
F	2	43	1	12	4	7	1	1	8	21
G	6	23	3	13	8	10	1	2	8	25
H	7	31	3	7	13	5	2	1	7	25
I	1	25	6	8	9	11	2	3	11	22

a NHS: National Health Service.

b FFT: Friends and Family Test.

c 0: unclassified.

d 1: respect for patient-centered values.

e 2: coordination and integration of care.

f 3: information and communication.

g 4: physical comfort.

h 5: emotional support.

i 6: involvement of family and friends.

j 7: transition and continuity.

k 8: access to care.

l 9: general.

### Establishing Ground Truth

Interrater agreement was determined on the manual coding, and the coding template was updated to check accuracy and improve the model by acquiring an updated corpus from the BoW and feature extraction. The interrater agreement was better for sentiment compared to theme. There was almost perfect agreement (0.81‐1.00) seen in 7 organizations. Trust A was the only organization with a substantial (0.61‐0.80) agreement with a Cohen κ of 0.79 (Table 3).

**Table 3.** Assessing the consistency of theme and sentiment ratings, and final accuracy via updated coding template.

Trust and category		Interrater reliability or Cohen κ	Accuracy before recoding (%)	Final accuracy (%)
A. Community and mental health				
	Theme	0.79	57	76
	Sentiment	0.82	76	82
B. Acute and inpatient				
	Theme	0.84	69	78
	Sentiment	1	87	91
C. Acute and inpatient				
	Theme	0.85	84	90
	Sentiment	0.91	87	94
D. Pediatrics				
	Theme	0.89	67	82
	Sentiment	1	72	82
F. General practitioner or community				
	Theme	0.80	64	77
	Sentiment	0.82	69	80
E. Acute and inpatient				
	Theme	0.96	*—^a^*	>75
	Sentiment	1	—	>75
G. Acute and inpatient				
	Theme	0.83	*—*	>75
	Sentiment	0.85	—	>75
H. Acute and inpatient				
	Theme	—	—	>75
	Sentiment	—	—	>75
I. Acute and inpatient				
	Theme	—	—	>75
	Sentiment	—	—	>75

a Not applicable given that the final 4 organizations reached thematic saturation following the coding efforts of the initial 5 organizations and therefore achieved an overall accuracy rate of 75% or higher.

**Figure 2.**
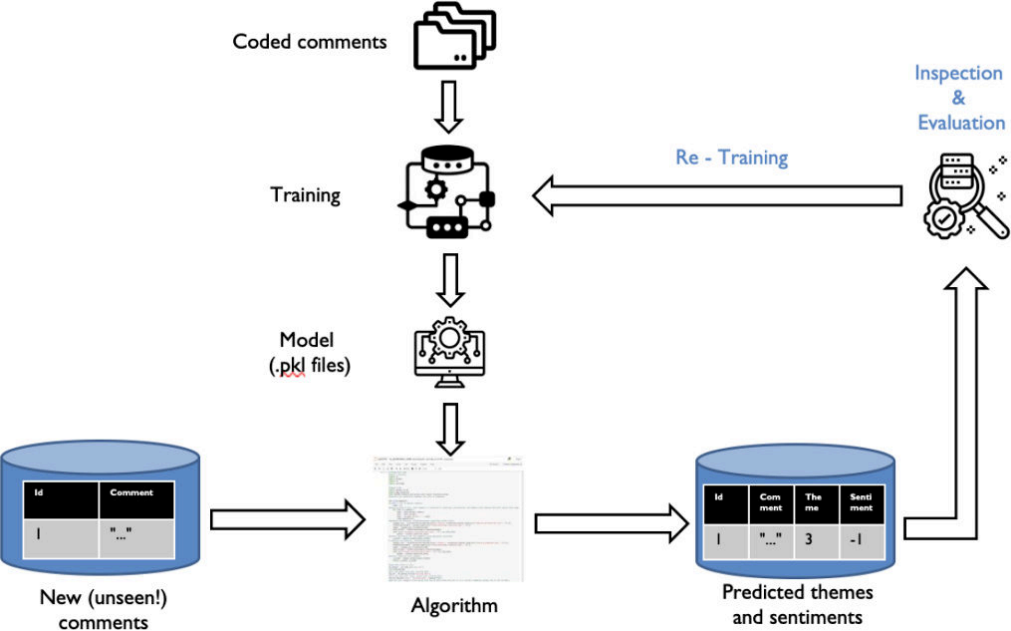
A pipeline created to develop and standardize the overall accuracy calculation before final deployment. Copyright owned by Imperial College Healthcare NHS Trust, 2025. All rights reserved.

Three trusts noted that sentiment prediction related to certain words was misclassified. This study’s team reviewed the performance and identified words that were culpable for this misclassification, for example, “nothing,” “everything,” and “OK.” These trusts were advised to manually code a subset of 500 comments with equally distributed sentiments, especially focusing on the misclassified words, along with comments that represented an equal or almost equal number of comments per themes or sentiments to obtain a balanced dataset. To determine the accuracy of the algorithm, a pipeline was developed and refined to standardize the entire process within all the organizations, as depicted in Figure 2.

The overall accuracy for theme and sentiment significantly improved after using a revised coded template. As trusts were added to the study sequentially, one by one, as part of the recruitment process, the BoW was also applied sequentially to the algorithm. Thematic saturation was achieved after analyzing and collating comments from a total of 5 trusts. This was observed when the algorithm was tested in 4 of the remaining trusts without the need to implement their respective coding output, and achieving an overall accuracy greater than or equal to 75%.

The final 4 organizations achieved an overall accuracy rate of 75% or higher, having reached thematic saturation following the initial coding efforts of the initial 5 organizations. As a result, as seen in Table 1, four versions of the model were deployed as new, labeled comments were collated from the different trusts that joined the program and deployed the algorithm. This iterative learning was carried out by adding randomly scrambled new unique words that the algorithm encountered in the BoW within its code. Each time, this word repository was completely randomized, and each word was treated individually by removing any linkage. As a result, it was not possible to know from which trust the word came from or if it was part of a positive or negative comment, with this study’s team holding the ground truth.

### Adaptations and Running Without Errors

**Figure 3.**
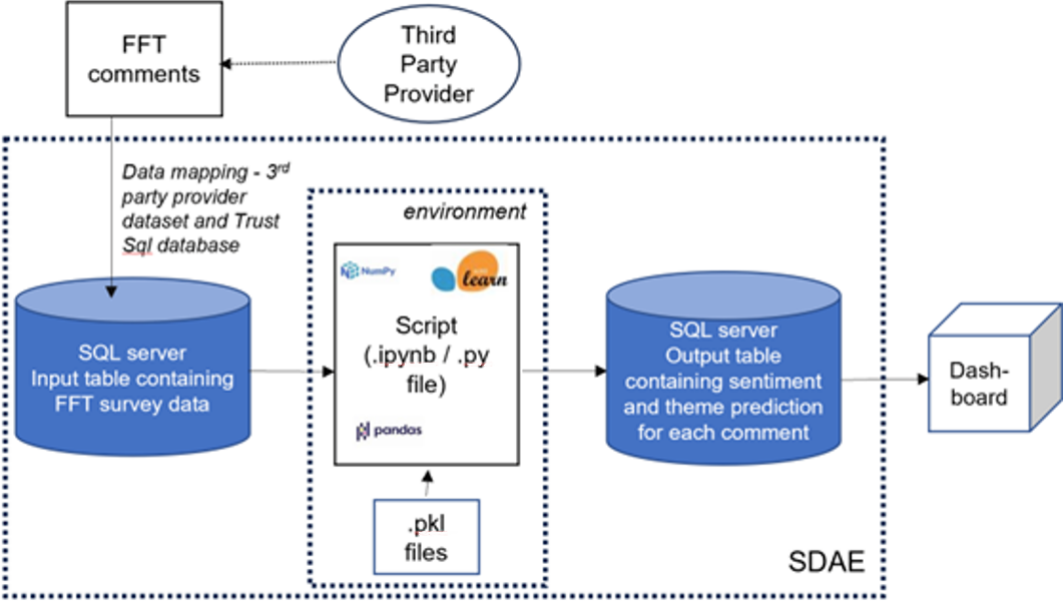
Final pipeline demonstrating steps required to deploy the final algorithm with FFT comments as input, prediction of themes and sentiment using pickle files generated from summative coding outputted to SQL database for use in dashboard visualization. FFT: Friends and Family Test. Copyright owned by Imperial College Healthcare NHS Trust, 2025. All rights reserved.

The algorithm script was adapted according to the agreements and requirements of the individual organization. The solution was tested in Jupyter Notebook (The Linux Foundation) for easier interaction with the code and checked for errors. The 2 most common errors encountered were “FileNotFoundError” which was a result of not specifying the correct path to a .pkl file, and “invalid column name,” as the code had not been adapted appropriately with the column names from the input table on the SQL server. Once errors were corrected, the final deployment consisted of the output with predicted themes and sentiment exported to a SQL database for integration into the dashboard visualization. This pipeline (Figure 3) was standardized after refinement with all participating organizations. Each trust automated its data flow based on the requirements defined by the frequency and volume of comments that the pipeline should be updated with.

For this purpose, user-friendly interfaces were designed and developed using the Flask Python library for the backend and HTML, CSS, and Bootstrap for the frontend. Functionalities of these interfaces included analyzing the accuracy of the algorithm by uploading a labeled dataset and retraining the algorithm with more coded data.

### Algorithm Refinement

Three hospitals noted that after manual inspection of the sentiment prediction, some FFT comments were being classified with an incorrect sentiment. We reviewed the performance of the algorithm and identified words that were making the algorithm perform incorrectly (eg “nothing,” “everything,” or “OK”). It was suggested that these hospitals should manually code a subset of 500 comments with equally distributed sentiments, especially focusing on these words, along with comments that represented an equal or almost equal number of comments per themes or sentiments, to obtain a balanced dataset. A code developed by this study’s team was provided to ensure the equal distribution of both themes and sentiments in the dataset.

### Multitagging

**Figure 4.**
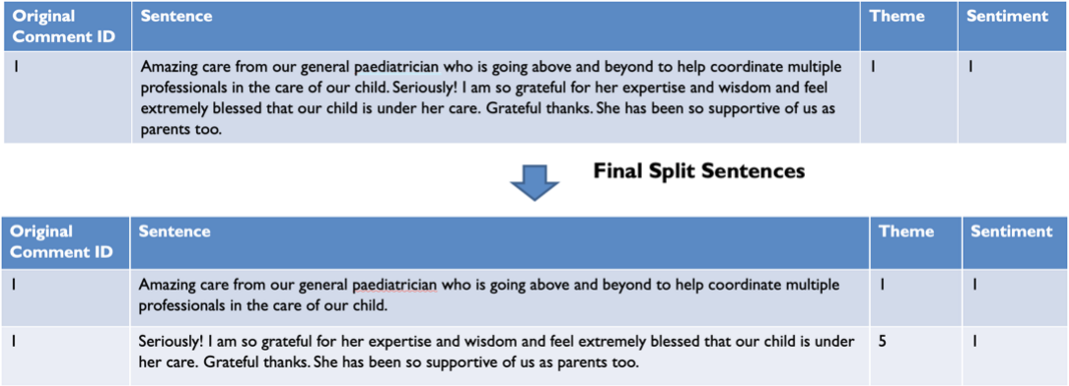
Example of how the sentencing approach works. The original comment is split into 2 subcomments, each classified according to a theme and a sentiment. The original comment identifier is retained to preserve the metadata of the comment. Copyright owned by Imperial College Healthcare NHS Trust, 2025. All rights reserved.

Potential refinements to the algorithm, particularly regarding the need to break long comments into sentences for trusts encountering lengthier narratives, such as those in mental health and community settings, were highlighted, and a multitagging method was devised. The multitagging approach involved assigning multiple themes from the NHS Patient Experience Framework to a single text document based on its content. Unlike the traditional single-label classification, where the FFT free-text is assigned to only 1 label, multitagging recognizes that comments may encompass various themes. This approach enables a more nuanced representation of the content’s complexity and captures multiple dimensions of meaning within the same document. The punctuation marks, words, and number of characters used to split the original sentence can be customized based on different requirements using Spacy (versions ≥3.7.2) and its English language model. As a default, sentences are split after a maximum of 75 characters, and each of these is analyzed individually (Figure 4). This can then be customized according to the requirements of the trust to define how the base text should be split (eg, by punctuation marks or specific words such as “however”). Based on this level of customizability, other trusts also implemented the solution.

### Redaction of Personal and Identifiable Information

Considerable variation was also observed in how trusts approached the redaction process, ranging from automated processes with subsequent manual checks to entirely manual methods. To address this inconsistency, a redaction algorithm capable of automatically detecting and masking personally identifiable information was devised. A redaction algorithm was developed using NLTK, Regex, and Presidio Python libraries to automatically detect and mask the personally identifiable information. The algorithm can be customized according to the requirements of the trust to define how and what data needs to be redacted.

### About CoP

**Figure 5.**
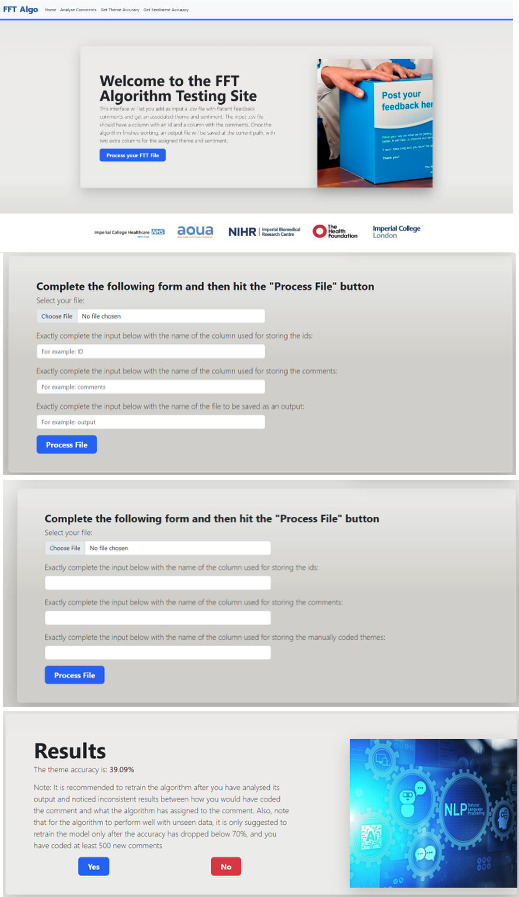
Interfaces developed by the community of practice to assist with regular algorithm maintenance. Copyright owned by Imperial College Healthcare NHS Trust, 2025. All rights reserved.

During the quarterly meetings, there was a high level of engagement from at least 80% of staff members representing the 9 trusts. This level of participation indicated strong interest and commitment to the CoP’s objectives. Additionally, feedback from participants revealed a positive perception of the CoP, with at least 90% reporting a sense of belonging and support within the community. This sense of camaraderie and mutual support contributed to the effectiveness of the CoP in addressing algorithm deployment challenges and driving improvements based on patient feedback. We addressed maintenance of the algorithm through the CoP by creating a standardized software that enables each hospital to check the accuracy of the algorithm and make changes as needed. Figure 5 shows the interfaces developed.

## Discussion

### Principal Findings

Using a test-and-iterate approach, this study deployed a text analytics algorithm to predict theme and sentiment from FFT free text data in 9 NHS organizations in England with different care settings, geographical locations, and demographics. Furthermore, this study highlighted the variation in technical capacity and resources among trusts, introduced and developed a framework, and standardized a data processing pipeline capable of supporting FFT text analytics bespoke to the organization’s needs. The key contributions of this study are: (1) the construction of technical infrastructure to support and run the algorithm effectively and being able to adapt the script for individual organizations; (2) the creation of master coding template with free-text data from different health care organizations to support algorithm iteration and improve overall accuracy for theme and sentiment; (3) the creation of pipeline to standardize the method of incorporating the algorithm for processing free-text data; and (4) foster collaborative nonsiloed working that encourages local ownership and share-learning.

### Addressing Data Management and Standardization Through IT Infrastructure

Data collection and analysis are central to this project. Achieving this required a robust data infrastructure with different components that collaboratively created an effective framework for research data management. This framework enhanced data accessibility by presenting information in a consistent, standardized, predictable, and accessible format while also strengthening data reliability through the automation of manual processes [22]. However, ensuring high-quality data demands a diverse set of information management skills, including data acquisition, storage, organization, retrieval, and long-term maintenance [23]. This project offered a valuable opportunity for trusts to gain expertise in this area by providing guidelines to streamline processes, define roles, and establish a resilient IT infrastructure.

### Creating a Template With Additional Organizational Specific Free-Text Data

Each health care setting possesses unique attributes, patient demographics, and linguistic nuances as demonstrated above. Algorithm testing was required to allow for customization and tailoring of the algorithm to accommodate these contextual variations. As part of a supervised learning approach, recoding was required to re-establish ground truth, and new words were incorporated into an expanded BoW to improve performance across various care settings. Literature suggests that the larger the training sets used, the higher the accuracy of the algorithms at identifying similar comments within the broader dataset, but trade-offs with time and human coding are necessary to ensure the method is resource-efficient [10]. However, the nature of patient experience vocabulary, due to the domain of patient feedback from free-text, is fixed in its nature, making it attractive data for supervised learning [24]. Therefore, it is possible to anticipate the meaning of various phrases and automatically classify the comments [25]. Thematic saturation was reached with 5 organizations, and no further coding was necessary. Just as the domain is fixed, the perspective of a patient feedback document is also fixed; there is limited vocabulary that is useful for commenting about health services [24]. Rastegar-Mojarad et al [26] also observed that a small (25%) vocabulary set covered a majority (92%) of the content of their patients’ comments, consistent with a study exploring consumer health vocabulary used by consumers [27]. This suggests that patients use specific vocabulary when expressing their experience within free-text comments.

In settings that were relatively fixed, that is, adult inpatient, the classifier trained strong reusable models that were evident from satisfactory accuracy. However, the accuracy of the model was lower in organizations delivering different services, such as pediatrics and mental health. This was to be expected, given that the language and type of words used in different care settings would vary from those found in an adult inpatient setting. The customization ensured that the algorithm aligns with the specific language and themes prevalent in the patient feedback of a particular health care organization. The accuracy for the theme improved in all the organizations demonstrating that while the algorithm may not initially be transferable, with some retraining and adaptations, it can easily be reused in different service settings. As part of the algorithm development process, cross-validation was applied to ensure the robustness and reliability of the model within the dataset used. In addition to this, we conducted external validation by incorporating patient comments from multiple trusts, ensuring that the model was exposed to a diverse range of data before declaring thematic saturation. This strategy allowed us to evaluate the model’s performance beyond a single dataset and ensured that it was not overfitting to any trust or setting. By including data from various trusts, we created a working external validation set, which will serve as the foundation for assessing the model’s generalizability in future implementations.

Furthermore, “respect for patient centered values” had the highest count in all organizations. This finding is similar to the literature around patient experience reporting in health care [28-32]. The reasons for this are twofold, first, primarily due to the large number of interchangeable words found within that theme, that is, “respect for patient-centered values, preferences, and expressed needs (cultural issues, dignity, privacy and independence, awareness of quality-of-life issues, and shared decision making).” Second, the overall domain of patient feedback is the health care system, and this study revealed that the content of reviews tends to focus on a small collection of aspects associated with this, as demonstrated by the themes used for text classification in the studies.

Through CoP participation, multitagging was used to assign multiple relevant tags or categories to individual sentences within a single FFT free-text comment. Instead of assigning a single label to the entire document, this technique involves breaking down text into sentences and assigning appropriate themes and sentiments to each based on its content, thereby enhancing customizability within mental health and community care settings. Furthermore, the CoP assisted in developing a redaction solution using Python libraries, which can be then customized according to the requirements of the trust to define how and what data needs to be redacted. These solutions can subsequently be adopted by other trusts as an improvement to their existing processes.

### Creating a Pipeline to Standardize the Algorithm Deployment Approach

As part of the model testing and to make the model more widely reusable, we created a data pipeline, which infuses and integrates BoW from different trusts to predict theme and sentiment at an accuracy greater than 75% when used in different trusts. This pipeline serves as a blueprint to stakeholders and facilitates understanding of all pieces of the pipeline among the team members and standardization when deploying the model. Compliance with regulations and governance is supported, and integration with DevOps practices enhances ML model integration into the software development lifecycle.

### Generating Cultural Change to Prioritize Patient Experience–Centered Care

The establishment of CoP can play a pivotal role in establishing a multi-disciplinary team of stakeholders from IT, business intelligence, patient experience, and quality improvement within each trust. Furthermore, the CoP can provide the necessary guidance to use the algorithm and assist in developing a dashboard for quality improvement projects [14], and sustained enthusiasm in the use of data and data analytics to improve the quality of care for patients and care givers [33]. This cultural transformation will result in increased organizational agility and responsiveness to patient needs, driving positive changes in care delivery practices [34].

### Limitations

The pickle files used in this study are specific to the version of Python and the libraries used to create them. Attempting to load a pickle file created with an older version of Python or with different library versions may result in compatibility issues. Newer versions of Python may have introduced changes to the pickle module or the libraries being pickled, which can result in errors when loading the file. To mitigate challenges with loading an outdated pickle file, updating the code that creates the pickle file, or using alternative serialization formats that are more version-independent can be considered. The model can be containerized using Docker with a requirements file containing all the required libraries and Docker specifying the environment setup and start-up operations. The Docker container can then be deployed to application hosting services, whether on cloud or on premises servers.

The limited vocabulary range is a significant issue faced by the BoW model. For example, if the model encounters an unfamiliar or rare but informative word that it has not yet seen during training, the BoW model will tend to ignore it. To mitigate this limitation, we trained the model on 5 of 9 trusts to ensure that the model had gathered a substantial BoW representation, further augmented by thematic saturation. Significant changes to patient demographics or significant changes, such as the COVID-19 pandemic, could reduce the performance due to unfamiliar words. Therefore, regular updates in the model would be required to keep it up to date. For this purpose, user-friendly interfaces can be designed and developed using the Flask Python library for the backend and HTML, CSS, and Bootstrap for the frontend. Functionalities of these interfaces included analyzing the accuracy of the algorithm by uploading a labeled dataset and retraining the algorithm with more coded data. The interfaces allow organizations to periodically check accuracy as part of a responsible AI strategy.

Given the limited analytical expertise and computational resources in many UK hospitals, complex models such as transformers and recurrent neural networks would be difficult to implement effectively. The SVM model was chosen for its simplicity, efficiency, and low resource demands, making it ideal for integrating into existing workflows. The focus was on maximizing the value of patient feedback data to improve care quality, without overwhelming the hospital’s IT infrastructure. We acknowledge that alternative lightweight ML methods, such as Decision Forests and XGBoost, offer similar efficiency and computational efficiency without the significant computational burdens often associated with deep learning models. Furthermore, modern deployment strategies, such as using pretrained architectures and lightweight deep learning models, can help mitigate the resource challenges typically associated with training deep learning models such as recurrent neural networks. As the hospital’s analytics capacity grows, more sophisticated models can be explored, but SVM provided a practical starting point for immediate, actionable insights.

The authors set out to illustrate the process of developing an NLP pipeline that originated in a single institution and has been iteratively refined for broader implementation. A strong technical knowledge base was essential to address variations in technical capabilities and IT infrastructure, as well as to introduce and enhance analytical skills that had been underused in some hospital trusts. However, to successfully implement the intervention within complex health care systems, it is crucial to understand how the intervention interacts with and influences various system components to achieve the desired outcomes. We are currently collaborating with participating trusts to establish a data flow that automates the integration of NLP outputs into a dashboard. Additionally, we are making algorithm improvements based on local priorities and needs to foster local ownership of the process.

### Responsible AI

As AI continues to advance, becoming increasingly integrated into health care systems, ethical considerations and technical challenges become increasingly intricate. For this reason, a key aspect of the project included providing tools and best practices to ensure that AI is being implemented responsibly. First, the developed user-friendly interfaces promote the ongoing monitoring of the accuracy of the algorithm, ensuring fairness and accountability of the version of the model that was deployed. Second, the establishment of CoP will play a critical role in fostering a culture of continuous improvement and supporting the transformation toward greater organizational agility, but also cultivates an environment where professionals can delve into the nuanced aspects of responsible AI. This transformation will enhance the responsiveness of health care systems to patient needs and drive positive changes in care delivery practices. Furthermore, by building local expertise and ownership, we aim to create a sustainable framework for ongoing use of data and analytics to improve the quality of care. This contributes to the development and refinement of ethical guidelines, playing a crucial role in shaping the responsible AI landscape.

### Future Directions

This study’s group is currently conducting a complex, multicomponent implementation designed to enhance operational efficiencies within the NHS by empowering frontline staff with visible and actionable insights derived from a patient feedback tool, which aligns with the NHS FFT redevelopment policy recommendations. This intervention aims to drive sustainable impact and foster a culture of patient-centered working by facilitating the identification and implementation of improvement opportunities. The CoP will play a vital role in achieving the broader goal of embedding a patient-centered working culture across the NHS. They will help sustain momentum for using patient feedback and data analytics to drive continuous improvements in care. This multifaceted approach ensures that the intervention not only has immediate operational benefits but also creates a lasting cultural shift toward patient-centered care.

Models such as BERT have the potential to offer deeper insights by capturing more complex linguistic patterns, which could be particularly valuable for understanding the subtleties in patient comments, especially in sensitive areas such as mental health. While we considered exploring these advanced models, we opted for a more straightforward approach during this phase of implementation. This decision was driven by the need for a method that is both practical and easily scalable across diverse health care settings. However, future research with a more experienced group, enriched by the extended knowledge gained through the CoP, could involve comparing transformer-based models such as BERT with the current approach to evaluate whether they provide significant improvements in accuracy and interpretability. Moreover, future work will explore the impact of different preprocessing strategies, such as retaining certain negations, expanding contractions, or adjusting tokenization rules (including experimenting with different n-gram configurations) based on contextual understanding. This would allow for a more nuanced approach, potentially improving the accuracy of sentiment classification, especially in cases where subtle cues are essential.

To enhance the interpretability of the model’s predictions, SHAP (Shapley Additive Explanations) and LIME (Local Interpretable Model-Agnostic Explanations) can be incorporated into the NLP pipeline. SHAP can explain that the words “excellent” and “care” contributed significantly to a positive prediction, while “waited” and “long” contributed to a negative prediction. This helps the health care team understand the key drivers behind feedback, allowing them to make targeted improvements. For a piece of patient feedback stating, “The doctor was kind, but the wait was too long,” LIME might perturb the sentence by removing words like “kind” or “wait” to see how the model’s prediction changes. If the model changes from positive to negative after removing the word “wait,” LIME would indicate that the term “wait” is highly important for determining the sentiment of the feedback. This could be an important step for trusts in improving transparency, which builds trust and helps the health care team make better-informed decisions when observing the dashboard.

### Conclusion

This study marks an advancement in harnessing free-text FFT data to gain valuable insights in health care settings through the creation of a robust supervised learning text analytics algorithm. The observed disparities in certain care settings were expected, considering the inherent differences in lexicon and terminology compared to the adult inpatient care setting where the algorithm was initially developed. Addressing these challenges involved additional coding and thorough testing under diverse scenarios. Through this iterative process, the accuracy and reliability of the algorithm were established to be robust and easy to use, fostering inter- and intraorganizational collaboration and promoting shared learning within the health care domain.

## Supplementary material

10.2196/60900 Multimedia Appendix 1 FFT capability review. FFT: Friends and Family Test.

## Abbreviations

AI: artificial intelligence
BoW: bag of words
CoP: community of practice
DPIA: Data Protection Impact Assessment
FFT: Friends and Family Test
FN: false negative
FP: false positive
IT: information technology
LIME: Local Interpretable Model-Agnostic Explanations
ML: machine learning
NHS: National Health Service
NLP: natural language processing
NLTK: Natural Language Toolkit
SHAP: Shapley Additive Explanations
SVM: support vector machine
TFIDF: term frequency-inverse document frequency
TN: true negative
TP: true positive
