# Supplementary material for: Leveraging AI to Drive Timely Improvements in Patient Experience Feedback: Algorithm Validation
Source: JMIR Med Inform. 2025 Jul 10;13:e60900. doi: 10.2196/60900 (PMC12270031; doi:10.2196/60900)
Supplement: Multimedia Appendix 1 [file medinform-v13-e60900-s001.pdf]

Section 1

FFT capability review

Domain	#	Question	Response	Total	Justification for inclusion
Patient Experience (Total= 18 points)	P1	In the last month, did you collect patient feedback across your services?	1. Yes, in all services (2)<br>2. Yes, in some services (1)<br>3. No (0 points)	2	To understand prevalence and spread of patient feedback data collection across the Trust.
	P2	How would you describe the level of staff in your Trust's patient experience team to provide dedicated support to frontline teams?	1. Very good (2 points)<br>2. Good (2 points)<br>3. Average (1 point)<br>4. Poor (0 points)<br>5. Very poor (points)	2	To understand whether the Trust PEx team have enough resources as is to provide support to frontline staff
	P3	Does your Trust have a dedicated team or staff member to analyse patient experience data in a timely manner?	1. Yes, every week (2 points)<br>2. Yes, every month (1 point)<br>3. Yes, every quarter (0 points)<br>4. Yes, on an ad-hoc basis (0 points)<br>5. No (0 points)	2	If the trust has a dedicated resource who has enough time to review and analyse FFT data in a timely manner, then the Trust has enough FFT capacity in Pex team.
	P4	Is patient experience data reported alongside other quality metrics across the Trust?	1. Yes, always (2)<br>2. Yes, sometimes (1)<br>3. No (0 points)	2	If FFT data is reported alongside other quality metrics, this indicates that Patient Experience is elevated and prioritised throughout the trust (a patient experience culture)
	P5	Are individual teams provided with their free-text patient experience data?	1. Yes, every week (2 point)<br>2. Yes, every month (1 point)<br>3. Yes, every quarter (0 points)<br>4. Yes, on an ad-hoc basis (0 points)<br>5. No (0 points)	2	To indicate micro-level use of free-text patient experience data
	P6	What % of frontline teams regularly review their patient experience data during their team meetings?	1. More than 80% (2 point)<br>2. Between 50 - 80% (1 point)<br>3. Less than 50% (0 points)	2	To indicate whether frontline staff are incorporating patient experience data review within their teams.
	P7	Is patient experience data displayed for patients in wards and service areas (e.g. using 'You Said, We Did' boards)?	1. Yes, always (2)<br>2. Yes, sometimes (1)<br>3. No (0 points)	2	To indicate whether the feedback loop is closed (i.e. patients - staff - patients)
	P8	Do you share and seek inputs on patient experience results through patient involvement groups or representatives?	1. Yes, always (2)<br>2. Yes, sometimes (1)<br>3. No (0 points)	2	To indicate whether there is already a strong culture of PPI (which would impact success of the PPI network proposed for this project).
	P9	In the last 3 months, is there evidence that patient experience metrics were discussed during Trust board meetings?	1. Yes, each month (2 points)<br>2. Yes, 1-2 times in the last three months (1 point)<br>3. No (0 points)	2	Visibility of FFT data and patient experience at board level, indicates how well patient experience is embedded throughout the organisation.
Quality Improvement (Total = 13 points)	Q1	Does your Trust have a quality improvement strategy that shares objectives with the organisational Patient Experience strategy?	1. Yes - briefly describe (1 point)<br>2. No - briefly describe (0 points)	1	To indicate whether quality improvement strategies for the Trust includes patient experience in addition to patient outcomes.
	Q2	Are there systematic QI methodologies (e.g. FlowCoach Academy, Model for Improvement, Lean Six Sigma, etc.) being used throughout your Trust?	1. Yes, always (2)<br>2. Yes, sometimes (1)<br>3. No (0 points)	2	To indicate level of experience within the Trust QI team
	Q3	How would you describe the level of staff in your Trust's QI team to provide dedicated support to frontline teams?	1. Very good (2 points)<br>2. Good (2 points)<br>3. Average (1 point)<br>4. Poor (0 points)<br>5. Very poor (points)	2	To understand whether the Trust QI team have enough resources as is to provide support to frontline staff
	Q4	To date, what proportion of your frontline staff have received training or support to develop their QI skills?	1. More than 50% (2 point)<br>2. Between 25 - 50% (1 point)<br>3. Less than 25% (0 points)	2	Frontline competencies around QI
	Q5	In the last month, what proportion of your frontline staff used quality improvement methods to undertake changes?	1. More than 80% (2 point)<br>2. Between 50 - 80% (1 point)<br>3. Less than 50% (0 points)	2	Day-to-day use of quality improvement methods by frontline teams.
	Q6	In the last 3 months, is there evidence that QI initiatives were discussed at Trust board meetings?	1. Yes, each month (2 points)<br>2. Yes, once in the last three months (1 point)<br>3. No (0 points)	2	Prioritisation of QI generally at board level
	Q7	Of the QI initiatives discussed at Trust board meetings, approximately what proportion were aimed at improving patient experience?	1. More than 50% (2 point)<br>2. Between 25 - 50% (1 point)<br>3. Less than 25% (0 points)	2	Prioritisation of QI initiatives to improve patient experience at board level. Often QI is focused on patient outcomes and not patient experience.
Digital Maturity (Total = 7 points)	D1	Are your patient experience survey free text responses systematically collected or transcribed digitally?	1. Yes + describe briefly (1 point)<br>2. No (0 points)	1	Indicates how much of the FFT free-text database is digitised for the algorithm to be deployed
	D2	Are your patient experience survey free-text responses shared digitally across the trust?	1. Yes, always (2)<br>2. Yes, sometimes (1)<br>3. No (0 points)	2	Indicates whether free-text FFT data is easily accessible and analysed electronically across the trust
	D3	Is the data you collect through patient experience surveys stored on a dedicated database / server that is accessible across the Trust?	1. Yes + describe briefly (1 point)<br>2. No (0 points)	1	Indicates whether FFT data is stored in one place / server or is fragmented across different places (this would have implications on overall visibility)
	D4	What type of visualisation software does your Trust use to report patient experience data?	1. Tableau, PowerBI, QlikView / QlikSense, etc. (2 points)<br>2. Excel workbooks (1 point)<br>3. None (0 points)	2	If data visualisation is automated, this indicates high levels of BI capability. 2 point for using automated softwares, 1 points if using excel because workbooks need to be manually updated and cannot be made real-time.
	D5	Is there a real-time link between the patient experience surveys database and the visualisation software (if one exists)	1. Yes + describe briefly (1 point)<br>2. No (0 points)	1	Indicates whether teams are already able to access FFT data without delays

© Imperial College Healthcare NHS Trust 2024. All rights reserved. For licensing enquiries contact nhsinfo@imperial.ac.uk

Section 2

IT scoping tool

FFT Scale, Spread, and Embed - IT Infrastructure Scoping Tool

Name of Trust	
Date of completion	

1. Key IT and data contacts

Please include the names of all key IT and data support contacts within the trust (e.g. IT infrastructure, BI, data analysis, etc.)

Name	Role / Job title	Email address

2. FFT Data Management and Visualisation

Who manages FFT data in the trust? (Fill w/ self-assessment questionnaire)

If other, please specify:

If managed by trust

Which database is used to store the FFT data?

If managed by external provider

What is the name of the external provider?

Key contact details of external provider:

Name	Role / Job title	Email address

What services do they provide? (e.g. data collection, data cleaning, visualisation and analysis)

Please describe the data transfer process, including how often the data is analysed

What dashboard or visualisation software is currently used to view FFT data?

3. IT infrastructure requirements:

What Frameworks, tools or IDEs do you use for python development / deployment? (Zeppelin, Jupiter, Text editor)

Do you need special approvals for installing libraries / software?

What infrastructure you use to deploy the model? (e.g. UK Cloud, Onprem, etc.)

3. FFT Data Structure

Please complete table below with the FFT questions currently asked in your trust.

Question	Response structure
Example: 1. Overall, how was your experience of our service	Scale 0-10
Example: 2. Please explain your answer	Free-text

© Imperial College Healthcare NHS Trust 2024. All rights reserved. For licensing enquiries contact nhsinfo@imperial.ac.uk

Section 3

NHS Patient Experience Framework

NHS

NHS Patient Experience Framework

In October 2011 the **NHS National Quality Board (NQB)** agreed on a working definition of patient experience to guide the measurement of patient experience across the NHS. This framework outlines those elements which are critical to the patients' experience of NHS Services.

- **Respect for patient-centred values, preferences, and expressed needs,** including: cultural issues; the dignity, privacy and independence of patients and service users; an awareness of quality-of-life issues; and shared decision making;
- **Coordination and integration of care** across the health and social care system;
- **Information, communication, and education** on clinical status, progress, prognosis, and processes of care in order to facilitate autonomy, self-care and health promotion;
- **Physical comfort** including pain management, help with activities of daily living, and clean and comfortable surroundings;
- **Emotional support** and alleviation of fear and anxiety about such issues as clinical status, prognosis, and the impact of illness on patients, their families and their finances;
- **Welcoming the involvement of family and friends,** on whom patients and service users rely, in decision-making and demonstrating awareness and accommodation of their needs as care-givers;
- **Transition and continuity** as regards information that will help patients care for themselves away from a clinical setting, and coordination, planning, and support to ease transitions;
- **Access to care** with attention for example, to time spent waiting for admission or time between admission and placement in a room in an in-patient setting, and waiting time for an appointment or visit in the out-patient, primary care or social care setting.

This framework is based on a modified version of the Picker Institute Principles of Patient-Centred Care, an evidence based definition of a good patient experience. When using this framework the NHS is required under the Equality Act 2010 to take account of its Public Sector Equality Duty including eliminating discrimination, harassment and victimisation, promoting equality and fostering good relations between people.

DH Department of Health

Section 4

Coding pack

Implementing the NLP algorithm in your trust

Three key steps need to be completed before deploying the NLP algorithm so that it can accurately analyse your FFT data:

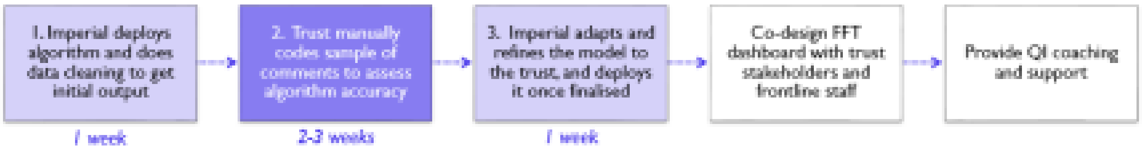

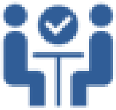

The project's natural language processing (NLP) algorithm is developed iteratively based on deployment within other trusts to ensure it is as robust as possible.

Each time the algorithm is deployed, we need to assess its accuracy by comparing it to a source of 'truth' – **the manual coding of a random sample of 500 FFT comments by two coders within the trust.**

This is a crucial and time-sensitive step as it ensures that the final algorithm can accurately analyze the FFT data, and we cannot continue with the project until this step is complete. **The coding process should take approximately 2-3 weeks to complete.**

Excel files in the coding pack

You will have received three excel files for the coding process. All file have been prepared with a 500 sub-sample of FFT free-text comments from your trust.

1. **Master coding file** – main coding file that will be used by Coder 1 to individually review comments and to review any coding differences

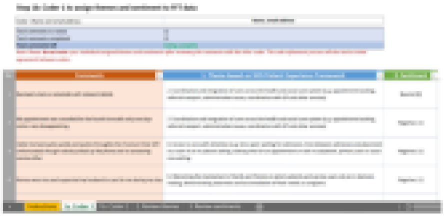

2. **Coder 2 coding file** – File that coder 2 can use to individually code comments. Once finished, the selected themes and sentiments should be copied over to the master file.

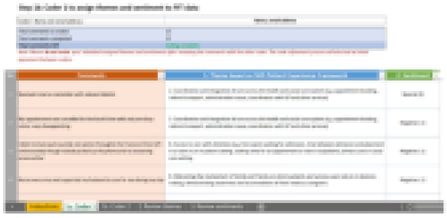

© Imperial College Healthcare NHS Trust 2024. All rights reserved. For licensing enquiries contact nhsinfo@imperial.ac.uk

Steps to code FFT free-text comments (1 of 3)

Agree on which two individuals within your trust will be doing the coding. We recommend selecting members of the Patient Experience team as they are most familiar with analysing FFT data for your trust.

Step 1: Over a week, code the first 100 comments together to ensure that both coders are analysing the FFT data in the same way. For each free-text comment, you will need to assign a **theme** based on the NHS Patient Experience Framework and a **Sentiment** (see slide 8).

Coder 1 should use the tab 1a. Coder 1, and coder 2 should use the tab 1b. Coder 2.

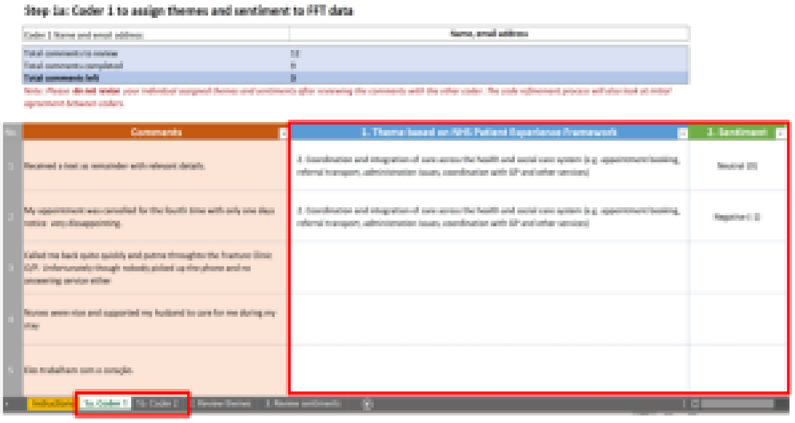

Steps to code FFT free-text comments (2 of 3)

Step 2: After coding the first 100 comments together, code the remaining 400 comments individually over a 1-2 week period.

Step 3: Copy Coder 2's individual coded comments into the master coding file.

Step 4: In the **"2. Review Themes" tab,** review any differences in coding of the themes between the two individuals. Filter for "yes" in the Differences column to only view comments that were coded differently. After discussion, recode the final agreed theme per comment.

Important: *Please do not revise your individual assigned themes after reviewing the comments with the other coder in the individual coding tabs. The code refinement process will also look at initial agreement between coders.*

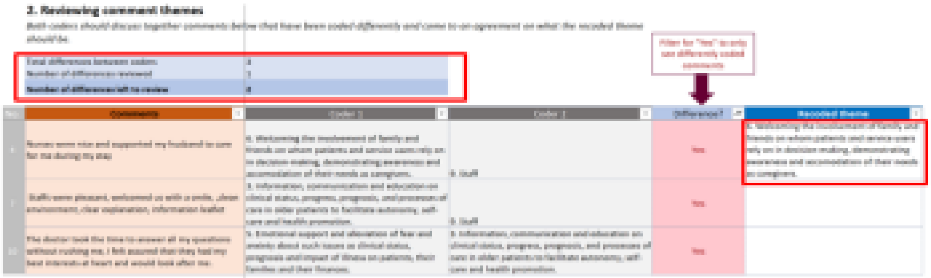

In this example, there are 3 comments with differences (comments 4,7,10), and the coders have recoded Comment #4 with Theme 6.

Steps to code FFT free-text comments (3 of 3)

Step 5: In the **"Review sentiments" tab,** review any differences in coding of the sentiments between the two individuals. Filter for "yes" in the Differences column to only view comments that were coded differently. After discussion, recode the final agreed sentiment per comment.

Important: *Please do not revise your individual assigned themes after reviewing the comments with the other coder in the individual coding tabs. The code refinement process will also look at initial agreement between coders.*

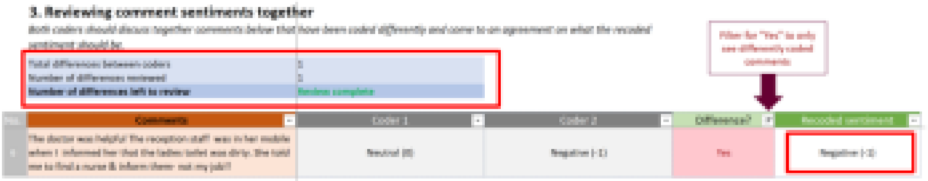

In this example, there was only 1 difference in sentiment coding (Comment 6) and it was recoded to be a negative sentiment. The sentiment review is complete.

© Imperial College Healthcare NHS Trust 2024. All rights reserved. For licensing enquiries contact nhsinfo@imperial.ac.uk

Frequently Asked Questions

1. What is the purpose of re-coding? *The initial algorithm was built on data from another Trust. In order to check its reliability, we need to assess for ground truth, which refers to the accuracy of the training set's classification for supervised learning techniques (a form of machine learning which is being used in this project).*
2. How many comments do we need to code? *500 stratified retrospective comments.*
3. Who needs to do the coding? *Two independent coders from the patient experience team (they must have experience of such coding and reading FFT free-text comments).*
4. What happens after the data has been coded? *We review the interrater agreement and the accuracy of the model to decide if the code needs to be further adapted to meet the FFT needs of your trust.*
5. What is the optimal inter-rater agreement? *We are aiming for 60-80%*
6. Why is the inter-rater agreement important? *Interrater agreement indices assess the extent to which the responses of 2 or more independent raters are concordant (consistent). This helps assess for accuracy of the supervised learning model as above.*
7. When the comment has more than one theme and sentiment, which theme and sentiment should be allocated? *Choose the one that the coder feels has more importance in the comment.*
8. Should the coders analyse the full FFT data sample together and not only just the first 100 comments? *We strongly recommend that you only review the first 100 comments together and do the rest individually to ensure the project can keep to timeline. This ensures both parties can provide support during the first stages of coding, with the additional ability to assess for interrater agreement.*
9. Do we need to do more coding in the future? *We would recommend coding a further 500 stratified comments every year to ensure the model remains consistent with patient comments and any changes are captured appropriately.*

© Imperial College Healthcare NHS Trust 2024. All rights reserved. For licensing enquiries contact nhsinfo@imperial.ac.uk
